# Integrated analysis of BSA-seq and RNA-seq identified the candidate genes for seed weight in *Brassica juncea*

**DOI:** 10.3389/fpls.2024.1458294

**Published:** 2024-12-03

**Authors:** Bin Yang, Liu Yang, Lei Kang, Liang You, Hao Chen, Huagui Xiao, Lunwen Qian, Yong Rao, Zhongsong Liu

**Affiliations:** ^1^ College of Agriculture, Hunan Agricultural University, Changsha, China; ^2^ Guizhou Institute of Oil Crops, Guizhou Academy of Agricultural Sciences, Guiyang, China; ^3^ Hunan University of Humanities, Science and Technology, College of Agriculture and Biotechnology, Loudi, China

**Keywords:** *Brassica juncea*, seed weight, BSA-seq, RNA-seq, candidate genes

## Abstract

**Introduction:**

*Brassica juncea* is a major oilseed crop of *Brassica*. The seed weight is one of yield components in oilseed *Brassica* crops. Research on the genetic mechanism of seed weight is not only directly related to the yield and economic value of Brassica juncea but also can provide a theory foundation for studying other Brassica crops.

**Methods:**

To map the genes for seed weight, the parental and F_2_ extreme bulks derived were constructed from the cross between the heavy-seeded accession 7981 and the light-seeded one Sichuan yellow (SY) of *B. juncea*, and used in bulk segregant sequencing (BSA-seq). Meanwhile, RNA-sequencing (RNA-seq) was performed for both parents at six seed development stages.

**Results:**

Our results showed that a total of thirty five SNPs were identified in thirty two genes located on chromosomes A02 and A10, while fifty eight InDels in fifty one genes located on A01, A03, A05, A07, A09, A10, B01, B02 and B04. The 7,679 differentially expressed genes were identified in developing seeds between the parents. Furthermore, integrated analysis of BSA-seq and RNA-seq data revealed a cluster of nine genes on chromosome A10 and one gene on chromosome A05 that are putative candidate genes controlling seed weight in *B. juncea*.

**Discussion:**

This study provides a new reference for research on *Brassica* seed weight and lays a solid foundation for the examination of seed in other *Brassica* crops.

## 1 Introduction

*Brassica juncea* is widely grown as an oilseed in China, India, Bangladesh, Ukraine, Canada and Australia (Carruthers et al., 2017; Kaur et al., 2020; Pal et al., 2021). Furthermore, this species is an important vegetable in China and is used as a condiment. *B. juncea* plants exhibit resistance to drought, poor soil, disease and insects and are adapted to hot, dry regions (Tian et al., 2017).

Seed weight, measured by thousand seed weight (TSW), is one of yield components in oilseed *Brassica* crops. Therefore, enhanced TSW is the direct way to increase yield (Gegas et al., 2010; Zhang et al., 2021; Zhou et al., 2022). Seed weight is reminiscent of seed size and seed fullness. Cytologically, seed development is regulated mainly by a combination of cell proliferation and cell enlargement (Sundaresan, 2005) and requires the synergistic growth of the embryo, endosperm and seed coat (Baud et al., 2008; Borisjuk et al., 2013). The maternal tissue can influence seed size through the regulation of transcription factor, ubiquitination, G protein and hormone signaling pathways, among others (Li and Li, 2016; Li et al., 2019). Seed weight is a quantitative trait controlled by multiple genes and influenced by genotype and environmental conditions (Yadava et al., 2012). The emergence of molecular markers, single-nucleotide polymorphism (SNP) chips and other technologies has greatly promoted the mapping of complex quantitative traits such as TSW (Dhaka et al., 2017; Li et al., 2014). The three QTLs (Quantitative Trait Loci) for TSW were identified on chromosome A07, C07 and C09 in *B. napus*, respectively (Quijada et al., 2006). The thirty four QTLs for TSW were also identified using DH populations, and SSR and AFLP markers (Basunanda et al., 2010). In addition, GWAS analysis identified the 320 SNPs linked with seed weight trait using 257 genotyped inbred rapeseed cultivars (Dong et al., 2018; Salami et al., 2024). However, the few loci for TSW have been cloned in *Brassica* species. Liu et al. successfully cloned the first TSW gene, *ARF18*, which controls both silique length and TSW (Liu et al., 2015). The gene *ARF18* promotes silique elongation, increases pod wall photosynthetic area, and therefore enhances TSW in rapeseed (Liu et al., 2015). Shi et al. have isolated the gene *BnaA9.CYP78A9D* which encodes a P450 monooxygenase that regulates the silique length by promoting cell elongation (Shi et al., 2019).Bulk segregant sequencing (BSA-seq) has been used to detect the allele frequency (AF) of SNPs and insertion-deletions (InDels) by constructing DNA pools of both parents and their F_2_ progenies with extreme values and thus identifying the associated genes (Gao et al., 2022). Using BSA-seq, Meng et al (Meng et al., 2023). located the candidate region associated with trichome traits and identified the candidate gene in *B. juncea.* Transcriptomic analysis using RNA-seq involves the study of gene expression at the RNA level. It is an important tool for studying the correlation between cell phenotype and gene function. Dynamic transcriptome analysis of varieties with contrasting seed sizes was used to identify the candidate genes associated with seed growth in *B. napus* and *B. rapa* (Geng et al., 2018; Niu et al., 2020). Recently, Shikha et al. performed comparative transcriptome analysis between two lines of *Brassica juncea*, small-seeded EG-2 and large-seeded PJ, at the initial stages of seed development and identified candidate genes regulating the cell cycle, cell wall biogenesis/modification, solute/sugar transport, and hormone signaling (Mathur et al., 2022). Moreover, transcriptomic analysis revealed that cell cycle-related genes play a key role in determining the seed size of *B. juncea* (Dhaka et al., 2022). However, the molecular regulatory mechanism of seed weight in *Brassica* species has not been fully elucidated.

In this study, we used the heavy- and light- seed accessions 7981 and Sichuan Yellow as parents to construct an F_2_ segregating population of *B. juncea*. Both parents and the bulks of the F_2_ progenies with extreme values were subjected to BSA-seq to identify the candidate regions associated with TSW in *B. juncea*. Meanwhile, differentially expressed genes (DEGs) were identified between the two parents via RNA-seq at different seed development stages. The combination of BSA-seq and RNA-seq identified candidate genes for determining seed weight in *B. juncea*.

## 2 Materials and methods

### 2.1 Plant materials and phenotyping

Based on the preliminary examination of global oilseed mustard (*Brassica juncea*) accessions grown in Changsha (short-day autumn sowing), Guiyang (short-day autumn sowing), Kunming (long-day summer sowing), and Xinjiang (long-day spring sowing) in two consecutive years (2018 and 2019), two accessions, 7981 and Sichuan Yellow, which showed phenotypic differences in thousand seed weight (TSW) across regions in different years, were chosen as materials. In this study, we named 7981 and Sichuan Yellow as the large seed (LS) and the small seed (SS), respectively.

The F_1_ hybrids was obtained by crossing 7981 with Sichuan Yellow, and self-pollinated to get F_2_ progeny. About 3,000 F_2_ individual seedlings were grown in Guiyang. The young leaves were collected from each individual and stored at -80°C for use. At maturity, a total of 1000 seeds from each individual plant were counted by a seed count instrument (CONTADOR, Pfeuffer, Germany), and then weighed by an electronic scale (CONTADOR, Germany). The mean of three replicates was taken as the measured value of TSW.

ANOVA was used to compare significant differences between the two seed lines.

### 2.2 Library construction and BSA-seq

Thirty individuals with extreme heavy TSW and the same number of individuals with extreme light TSW were selected from F_2_ progeny and used for construction of F_2_ extreme bulks. Ten individual plants from each parent were used for construction of the parental bulks. The genomic DNA was extracted from each bulk using the modified cetyltrimethyl ammonium bromide (CTAB) method (Clarke, 2009). The purity and integrity of the DNA were detected using a NanoDrop instrument (NanoDrop, DE) and agarose gel electrophoresis, respectively. The DNA was quantified by a Qubit Fluorometer (Thermo Fisher, MA). The libraries were prepared according to the standard protocol of Illumina and sequenced on an Illumina HiSeq2500 platform (Illumina, CA).

The clean reads obtained after removing the adapter from the raw reads were aligned to the *B. juncea* reference genome (*Brassica juncea* (L.) Czern & Coss, http://117.78.45.2:96/genomes/) using BWA (v. 0.7.15-r1140, Burrows−Wheeler-Aligner) (Li and Durbin, 2009). SAMtools (v. 1.3.1) was used to remove duplicates (Li et al., 2009). Then, the variant calling of SNPs and InDels was performed with GATK (McKenna et al., 2010). ANNOVAR was used to annotate the filtered SNP calls, and a high-quality SNP set was ultimately obtained (Wang et al., 2010).

### 2.3 Identification of candidate genes for TSW by analysis of allelic frequency difference

Based on genotyping results, SNPs with homozygous differences between two parents were screened. Then, the SNP-index (SNP frequency) of each F_2_ bulk was calculated in each screened SNPs using the parent as the reference. In order to reduce the impact of sequencing and mapping errors, the SNPs were filtered according to the SNP-index results, the filtration criteria are as follows: 1) the SNPs with SNP-index less than 0.3 and SNP depth less than 7 in both F_2_ individual pools, 2) a site where the SNP-index of F_2_ progenies is missing, filters it out. To directly reflect the distribution of SNP-index on chromosomes of each F_2_ individual, the distribution of SNP-index on chromosomes was mapped. The 1Mb is selected as the window and 1kb as the step size. The average SNP-index in each window is calculated to reflect the SNP-index distribution of the two F_2_ bulks. After calculating the SNP-index of each F_2_ individual in each SNP sites, the △(SNP-index) value of each variant was calculated via QTLseqr (R package). Then, the 1,000 permutation tests were performed and the 95% confidence interval (CI) selected as the threshold for screening the candidate region of TSW. The regions with △SNP-index) above the 95% CI were identified as candidate regions for TSW.

As above, based on genotyping results, the InDels of homozygous differences between two parents were screened. The missing sites of F_2_ bulks were filtered out to reduce the impact of sequencing and alignment errors. Then the InDel-index and △(InDel-index) were calculated using the above methods, and the regions with △(InDel-index) above the 95% CI were identified as candidate regions for TSW.

According to the annotation information of SNPs and InDels in candidate regions, sites causing stop loss or stop gain, non-synonymous mutations, variable splicing sites or frameshift mutations were preferentially selected, and the genes having these sites were selected as candidate genes.

### 2.4 RNA-seq library preparation and sequencing

The developing seeds were harvested for RNA-seq from both parents at 15, 20, 25, 30, 35 and 40 days after pollination (DAP), referred to as stages S1, S2, S3, S4, S5 and S6, respectively. Three biological replicates were performed for each seed sample. The mRNA libraries were constructed from high-quality RNA samples using an NEBNext Ultra Directional RNA Library Prep Kit (NEB, UK) according to the manufacturer’s instructions and sequenced on a HiSeq 4000 (Illumina, San Diego) using a paired-end run (2 × 150 bp). The raw reads generated were filtered to remove adapter, poly-N and low-quality reads. Then, the clean paired-end reads were aligned to the above *B. juncea* reference genome using HISAT2 (v.2.1.0) with default parameters (Kim et al., 2015).

### 2.5 Identification and annotation of DEGs

The gene expression level was estimated by HTSeq (v.0.6.1), and an FPKM greater than 1 was used as a threshold for determining gene expression (Trapnell et al., 2012). Differentially expressed genes (DEGs) between 7981 and Sichuan yellow at six seed developmental stages were identified using the DESeq2 package (Love et al., 2014). A DEG with a log2 ratio > 0 in the read count between two libraries was considered as upregulated expression, while a log2 ratio<0 was considered as downregulated expression. The DEGs were annotated by Gene Ontology (GO) functional enrichment using GOseq (v.2.12) (Young et al., 2010) and Kyoto Encyclopedia of Genes and Genomes (KEGG) functional enrichment using KOBAS (v. 2.0) (Kanehisa et al., 2008). DEGs were considered as significantly enriched if the adjusted *P* value was <0.05.

### 2.6 qRT−PCR validation of the DEGs

Differentially expressed genes were subjected to qPCR analysis using the Bio-Rad CFX96 Real-Time PCR System with a two-step protocol after total RNA extraction, quantification, qualification, and first-strand cDNA synthesis. The primers used for the qRT−PCR analysis is shown in **Supplementary Table S1**. The reaction program included an initial denaturation at 95°C for 30 s, followed by 40 cycles of denaturation at 95°C for 10 s and annealing and extension at 60°C for 30 s. Each sample was run with three biological replicates. The expression level of the actin gene was used as an internal control. The relative expression levels of each gene were calculated with the 2^–ΔΔCt^ method (Livak and Schmittgen, 2001).

## 3 Results

### 3.1 Seed phenotype of two *B. juncea* parental accessions

**Figure 1:**
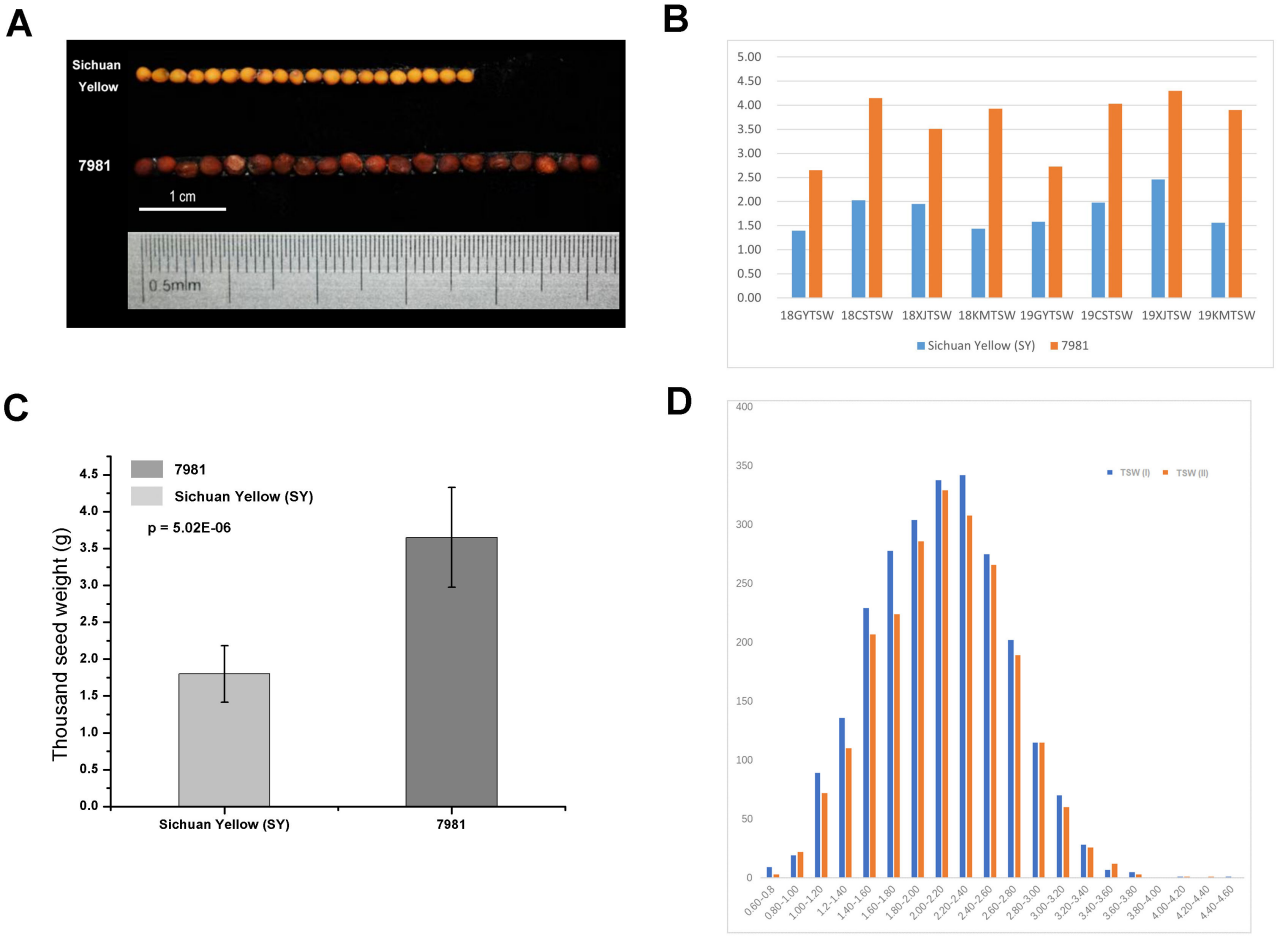
The phenotypic comparisons between the *Brassica juncea* accessions 7981 and Sichuan Yellow. **(A)** Comparisons in seed size and color in matured seeds of 7981 and Sichuan Yellow. **(B)** the TSW of two accessions grown in four geographical sites at two consecutive years (2018, 2019). e.g. the 18GYTSW in x axis means the seeds collected in Guiyang at 2018. **(C)** Comparison in TSW of matured seeds between 7981 and Sichuan Yellow. The measurement data are shown with standard error bars from four geographical sites at two years. The data are the means ± SE (n = 8). *p* value denotes a significant difference between two lines in TSW. **(D)** The frequency distribution in TSW of F_2_ individual plants.

The seed phenotype of both parents 7981 and Sichuan yellow (SY) was shown in **Figure 1**. As shown in **Figure 1B**, TSW of 7981 grown at four sites was heavier than that of Sichuan Yellow at two consecutive years. The mean TSW of Sichuan Yellow is 1.8g whereas that of 7981 is 3.7g over four sites in two years (**Figure 1C**). In addition, the seed size also exhibited significant differences between the parents (**Figure 1A**). **Figure 1D** showed the distribution of F_2_ progeny in TSW.

### 3.2 Detection of SNPs and InDels by BSA-seq

BSA-seq generated raw data of a total of 126,953,330,700 bp. After quality control, 125,289,937,200 bp of clean data were obtained. The guanine cytosine (GC) content of these clean reads ranged from 38.06% to 39.75%, and the quality of the sequencing data was high (Q20 ≥ 96.31%, Q30 ≥ 90.22%) (**Supplementary Table S2**). The clean reads were subsequently mapped against the reference genome, with a mapping rate ranging from 97.27% to 99.11% and an average coverage of 11× for both parents and approximately 40× for two F_2_ bulks. The degrees of coverage of 1× and 4× were 88.85% and 77.79%, respectively (**Table 1**).

**Table 1:** Quality statistics of mapping with the reference genome for BSA-seq.

Sample	Mapped reads	Total reads	Mapping rate (%)	Average depth (X)	Coverage at least 1X (%)	Coverage at least 4X (%)
7981	78,439,304	80,024,838	98.02	11.75	88.85	77.79
Sichuan Yellow	88,373,484	89,167,750	99.11	11.9	97.71	91.4
LS_bulk	308,440,316	317,095,674	97.27	39.71	98.65	98.05
SS_bulk	341,588,558	348,977,986	97.88	50.04	98.53	97.92

A total of 4,801,126 SNPs and 1,063,911 InDels were called out using GATK 3.8 (**Supplementary Table S** **3**). Among them, 576,970 SNPs and 38,158 InDels were mapped to the exon regions using ANNOVAR, including 3,715 SNPs and 1,300 InDels in “Stop gain”, 842 SNPs and 144 InDels in “Stop loss”, 338,742 SNPs in “Synonymous”, 230,855 SNPs in “Nonsynonymous”, 11,159 InDels in “Frameshift deletion”, 10,136 InDels in “Frameshift insertion”, 7,868 InDels in “Nonframeshift deletion”, and 7,551 InDels in “Nonframeshift insertion” (**Supplementary Table S3**).

### 3.3 Identification of candidate genes for TSW in *B. juncea*

**Figure 2:**
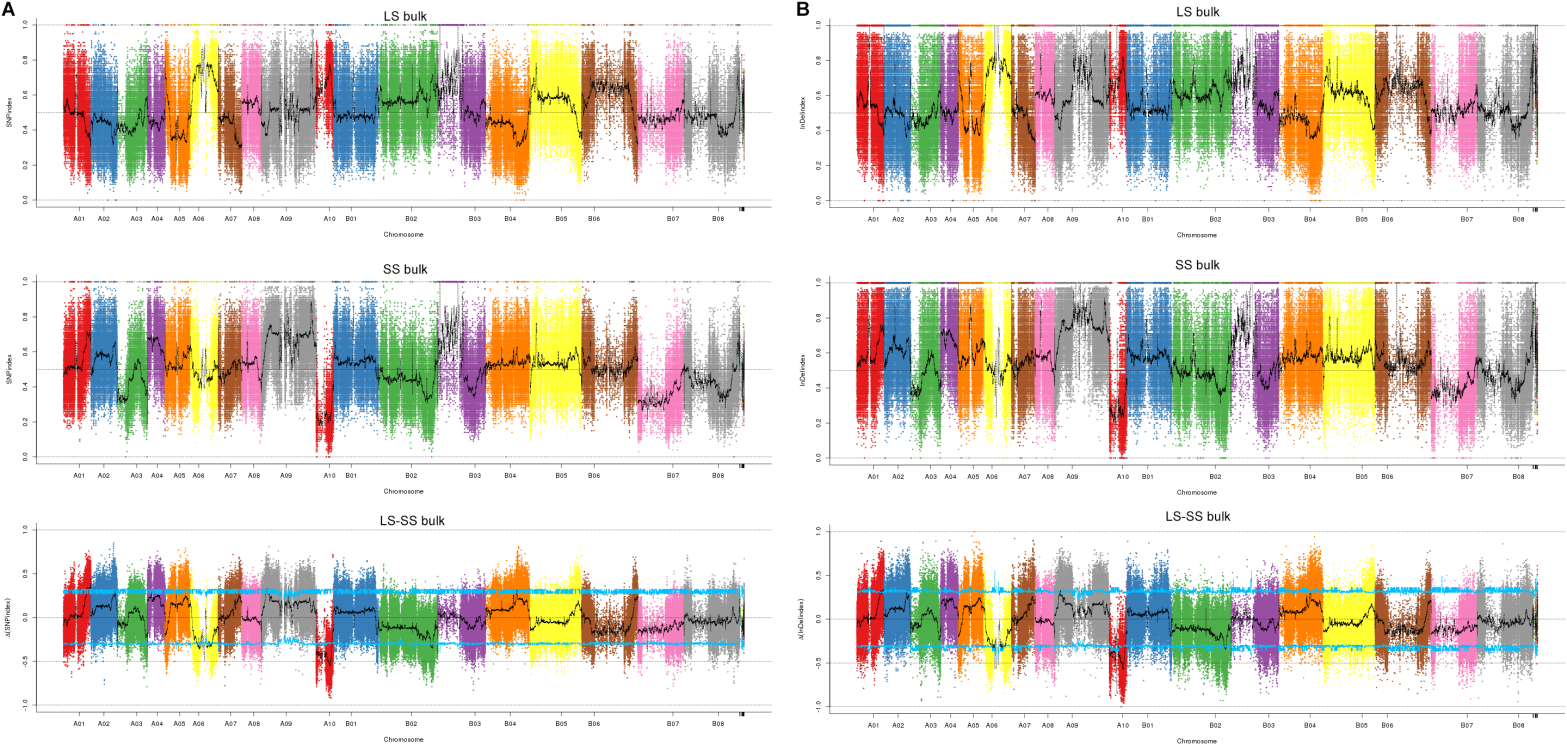
Distribution of SNP and InDel association values on chromosomes. **(A)** the distribution of SNPs. **(B)** the distribution of InDels. LS bulk means distribution of SNP- or InDel-index values of heaviest seed bulk on chromosomes, SS bulk means distribution of SNP- or InDel-index values of lightest seed bulk on chromosomes, LS-SS bulk means distribution of △ (SNP- or InDel-index) value on chromosomes, where the blue line represents the 95% CI threshold.

From the 822,583 SNPs with homozygous differences between two parents, we identified 817,134 SNPs with SNP-index values more than 0.3 and depths more than 7. The Manhattan map showed the distribution of SNP-index of each F_2_ bulk (**Figure 2A**). 120 SNPs on chromosomes A01, A02, A09, A10, B02 and B07 were identified with the △(SNP-index) value above the 95% CI (**Supplementary Data Sheet S1**). Annotation of these SNPs revealed that thirty-one SNPs were located in exons and these SNPs were distributed in chromosome A10, including eight synonymous mutations (**Table 2**, **Supplementary Data Sheet S1**). These eight nonsynonymous mutations were in six genes (*BjuA10g14470S*, *BjuA10g16450S*, *BjuA10g18390S*, *BjuA10g18960S*, *BjuA10g20540S* and *BjuA10g23440S*) (**Table 1**). Among these genes, *BjuA10g16450S*, *BjuA10g18960S*, *BjuA10g20540S* and *BjuA10g23440S* were annotated as a PIWI domain, a BolA-like protein, a PPR and a histidine kinase, respectively (**Table 1**). In addition, a total of 32 genes (30 genes in chromosome A10 and 2 genes in chromosome A02) harboring 35 significant SNPs were identified as candidate genes according to the annotation of candidate region of SNPs (**Supplementary Data Sheet S2**).

**Table 2:** The eight SNPs and four InDels within the candidate region and located in exon.

SNP	Chr.	Pos.	REF	ALT	Type of mutation	Associated gene	Gene description
1	A10	14360757	G	C	nonsynonymous SNV	BjuA10g14470S	–
2	A10	15706305	G	A	nonsynonymous SNV	BjuA10g16450S	PF02171:Piwi domain PF16488:Argonaute linker 2 domain
3	A10	16745753	G	A	nonsynonymous SNV	BjuA10g18390S	PF14365:Domain of unknown function (DUF4409)
4	A10	17075651	A	G	nonsynonymous SNV	BjuA10g18960S	PF01722:BolA-like protein
5	A10	17858118	G	A	nonsynonymous SNV	BjuA10g20540S	PF13041:PPR repeat family PF01535:PPR repeat
6	A10	19294411	G	C	nonsynonymous SNV	BjuA10g23440S	PF02518:Histidine kinase
7	A10	19294420	T	A	nonsynonymous SNV	BjuA10g23440S	PF02518:Histidine kinase
8	A10	19294448	G	C	nonsynonymous SNV	BjuA10g23440S	PF02518:Histidine kinase
InDel	Chromosome	Pos	REF	ALT	Type of mutation	Related gene	Gene description
1	A05	26913983 26913988	CCTCCC	–	nonframeshift deletion	BjuA05g30130S	–
2	A10	7749369	–	CC	frameshift insertion	BjuA10g08890S	PF00215:Orotidine 5’-phosphate decarboxylase/HUMPS family|PF00318:Ribosomal protein S2
3	A10	14426783	–	A	frameshift insertion	BjuA10g14570S	PF13041:PPR repeat family
4	A10	18666735	–	CAG	nonframeshift insertion	BjuA10g22200S	PF02045:CCAAT-binding transcription factor (CBF-B/NF-YA) subunit B

– means no data detected.

Similarly, 687,851 InDels were filtered out from 694,636 InDels. After calculating the InDel-index and △(InDel-index), the distribution of each bulk was presented in Manhattan map (**Figure 2B**). According to the △(InDel-index), 302 InDels were identified with the △(InDel-index) value above 95% CI (**Supplementary Data Sheet S3**). The annotation showed that four InDels were located within exons, including one frameshift insertion, one nonframeshift insertion and two nonframeshift deletions (**Table 2**, **Supplementary Data Sheet S3**). Among these InDels, three were located in three genes, namely, *BjuA10g22200S* (a CCAAT-binding transcription factor-encoding gene), *BjuA10g14570S* (a PPR repeat protein-encoding gene) and *BjuA10g08890S* (a ribosomal protein S2-encoding gene) (**Table 2**). The candidate gene analysis showed that fifty eight InDels significantly associated with TSW were distributed on chromosomes A01, A03, A05, A07, A09, A10, B01, B02, and B04, and located in 51 candidate genes (**Supplementary Data Sheet S2**).

### 3.4 Dynamic seed transcriptomic comparison between both *B. juncea* parents

To explore the seed weight-related genes in *B. juncea*, the seed transcriptomes were dynamically analyzed via RNA-seq. More than 1.66 ×10^9^ raw reads were generated from thirty-six libraries, with an average of 6.79 Gb of clean reads for each sample and a Q30 of 92.7% across all samples (**Supplementary Table S4**). Mapping of the clean reads to the SY reference genome yielded a percentage of 90.89% (**Supplementary Table S4**).

### 3.5 Differentially expressed genes shared at various seed development stages between *B. juncea* parents

**Figure 3:**
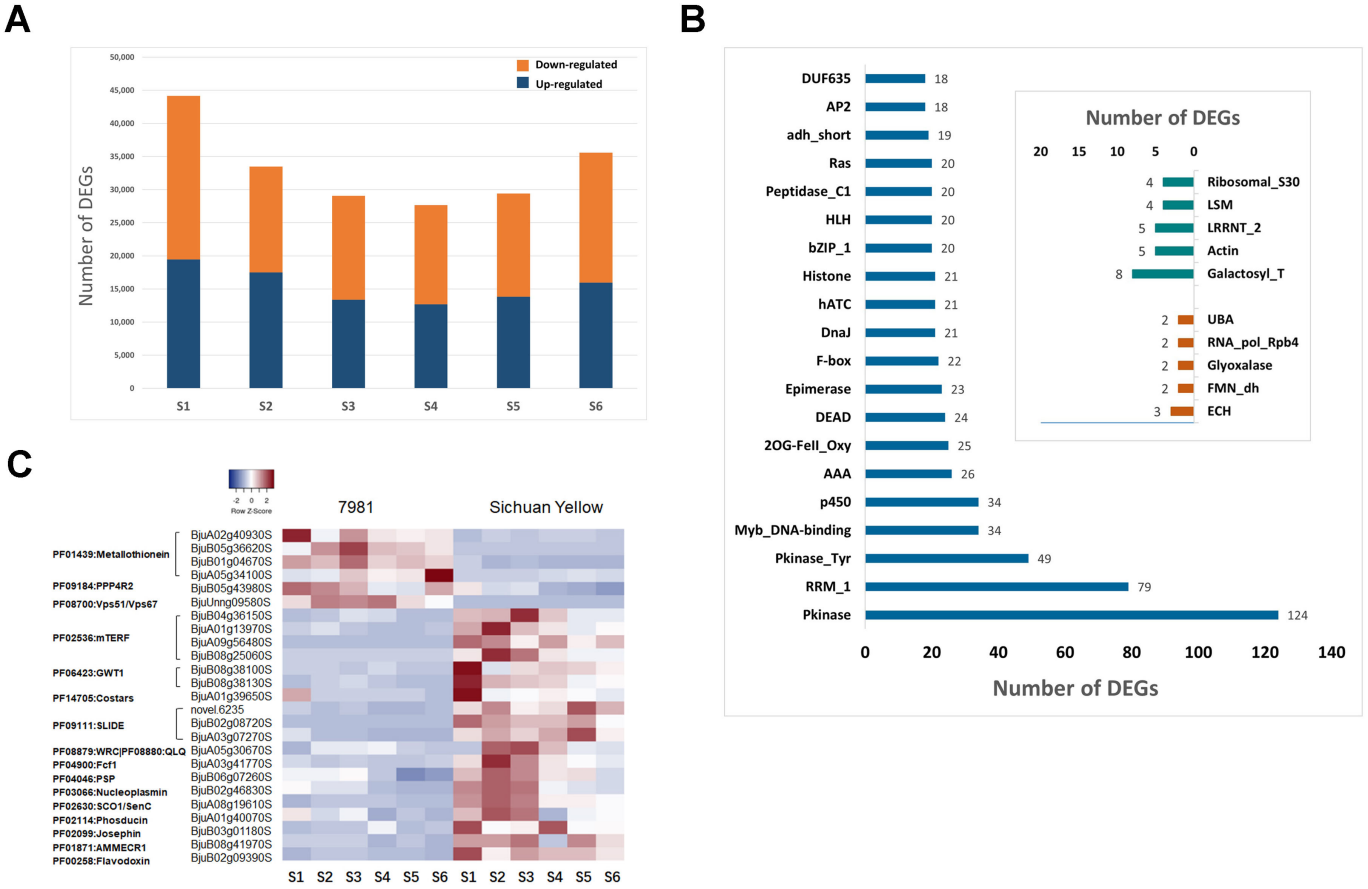
Analysis of differentially expressed genes (DEGs) at six seed development stages. **(A)** The number of up- and down-regulated DEGs. **(B)** The Transcription factor gene family of the most DEG. **(C)** The heatmap of DEGs.

The genes that were differentially expressed were compared between both parents for each seed development stage. The highest number (44,176) and the lowest number (27,678) of DEGs were found between 7981 and SY at 15 and 30 DAP, respectively (**Supplementary Table S5**). 33,478, 29,078, 29,421 and 35,581 DEGs were detected at 20, 25, 35 and 40 DAP (**Supplementary Table S5**). The number of up- and down-regulated DEGs in the seeds at each stage between both parents is shown in **Figure 3A**.

A total of 7,679 DEGs were shared with each seed developmental stage (**Supplementary Data Sheet S4**), among which 2,278 belonged to the transcription factor gene families, with the top 10 TFs being Pkinase, RRM_1, Pkinase Tyr, Myb_DNA-binding, p450, AAA, 2OG-FeII_Oxy, DEAD, Epimerase and F-box (**Figure 3B**). Except for these DEGs classified as TF family, the 2,792 of 7,679 DEGs were also annotated. Among these, there were 6 DEGs identified up-regulated and 19 DEGs down-regulated in accession 7981 at six development stages (**Figure 3C**). Gene annotation showed that the 6 DEGs were PF01439:Metallothionein and 2 DEGs annotated in PF09184:PPP4R2 and PF08700:Vps51/Vps67, and the 19 down-regulated DEGs were PF02536:mTERF, PF06423:GWT1, PF14705:Costars, PF09111:SLIDE, PF08879:WRC|PF08880:QLQ, PF08511:COQ9, PF04900:Fcf1, PF04046:PSP, PF03066:Nucleoplasmin, PF02630:SCO1/SenC, PF02114:Phosducin, PF02099:Josephin, PF01871:AMMECR1 and PF00258:Flavodoxin, respectively (**Figure 3C**).

**Figure 4:**
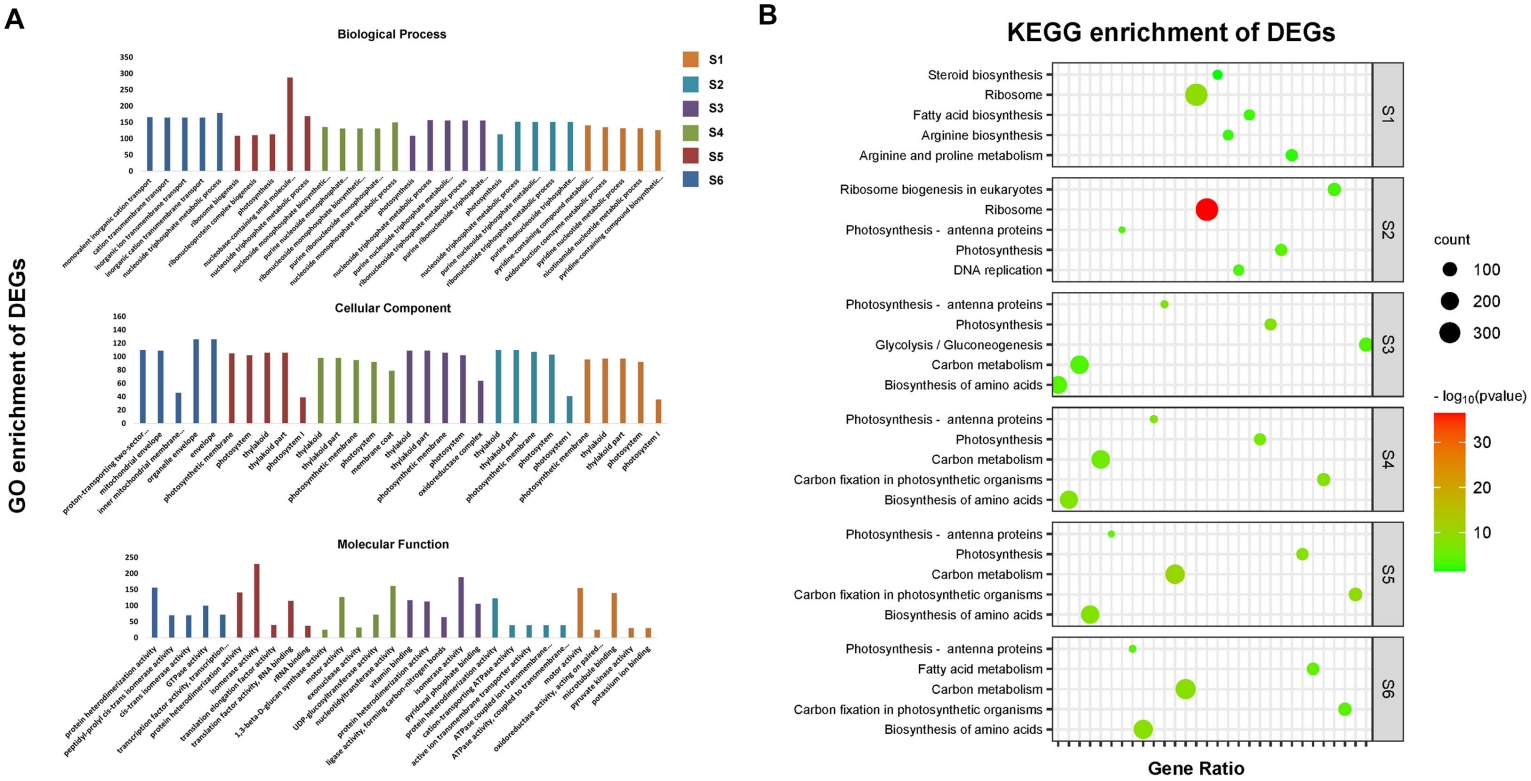
The Gene ontology classification and KEGG enrichment of DEGs. **(A)** The top 5 GO items most significantly enriched by DEGs at six seed development stages. **(B)** The top 5 KEGG items most significantly enriched by DEGs at six seed development stages.

To investigate the biological processes and pathways, these DEGs are involved in the Gene Ontology (GO) annotation and KEGG annotation were conducted (**Figure 4**). The top 5 most enriched GO and KEGG items by DEGs in six compared groups were further analyzed. A total of 61 GO items identified were classified into three categories of biological process, cellular component and molecular function (**Figure 4A**). In the biological process class, most of the DEGs were associated with ion transmembrane transport-, nucleotide-, purine-, pyridine- or ribosome-related processes. Among the cellular component terms, the GO terms “envelope”, “membrane coat”, “mitochondrial envelope”, “oxidoreductase complex”, “photosystem”, and “thylakoid” were most enriched. Among the molecular function terms, the significantly overrepresented items were protein heterodimerization activity, isomerase activity and motor activity (**Figure 4A**). KEGG annotation indicated that most of the DEGs were enriched in the ribosome, fatty acid biosynthesis, arginine biosynthesis and arginine and proline metabolism pathways 15~20 DAP, while the five pathways associated with the most enriched genes were photosynthesis, photosynthesis-antenna proteins, biosynthesis of amino acids, glycolysis/gluconeogenesis, carbon metabolism, and carbon fixation 25-40 DAP (**Figure 4B**).

### 3.6 Putative candidate genes for TSW in *B. juncea*

**Figure 5:**
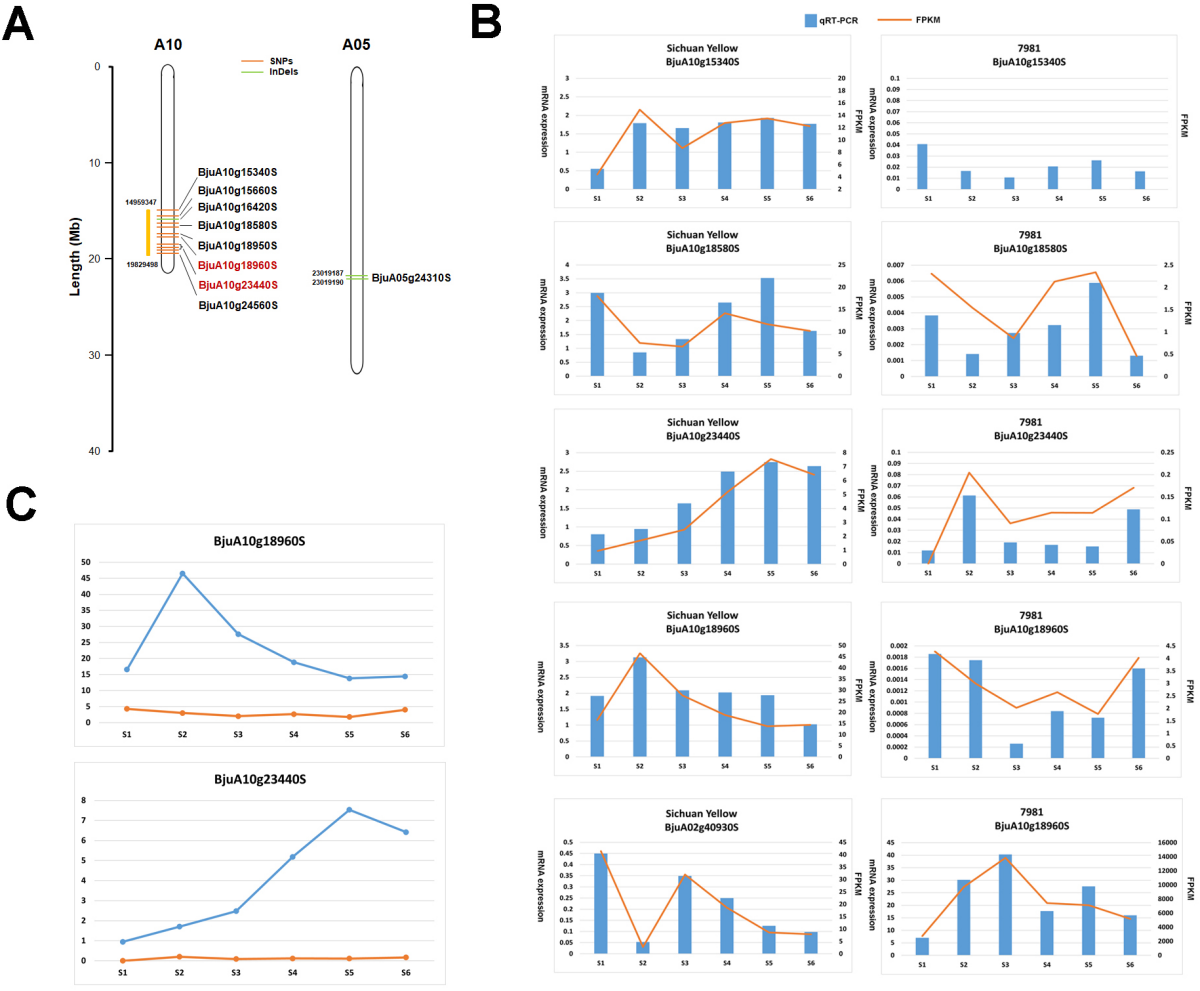
Candidate gens for thousand-seed weight (TSW) identified by BSA-seq and RNA-seq analysis. **(A)** The genomic location of candidate genes. **(B)** The expression level of candidate genes. **(C)** The expression of two most likely candidate genes for TSW.

As mentioned above, the BSA-seq analysis reveals that thirty five significant SNPs located in thirty two genes and fifty eight significant InDels in fifty one genes were associated with TSW. Meanwhile, the transcriptome profiling showed that there were 7,679 DEGs between two accessions contrasting in seed weight. Integrated analysis of the above BSA-seq and RNA-seq data identified nine genes (*BjuA10g15340S*, *BjuA10g15660S*, *BjuA10g18580S*, *BjuA10g18950S*, *BjuA10g18960S*, *BjuA10g23440S*, *BjuA10g24560S*, *BjuA05g24310S*, *BjuA10g16420S*) which showed difference in structure and/or expression level (**Table 3**, **Figure 4A**). Among these genes, *BjuA10g18960S* and *BjuA10g23440S* harbored nonsynonymous SNPs. The former encodes the BolA-like protein, while the latter encodes the HSP90-like ATPase (**Table 3**, **Figure 5A**). Notably, the expression of *BjuA10g18960S* and *BjuA10g23440S* was significantly higher in Sichuan Yellow than in 7981 (**Figure 5C**), which was confirmed by qRT−PCR analysis (**Figure 5B**).

**Table 3:** The candidate genes related to TSW identified by analysis of BSA-Seq and RNA-Seq.

Candidate SNPs/InDels	Gene_id	Variant	Chr.	Pos.	Ref	Alt	Log2FC	Gene description
SNPs	*BjuA10g15340S*	Down stream	A10	14959347	T	C	-8.05	––
	*BjuA10g15660S*	Up stream	A10	15187411	G	A	-1.83	––
	*BjuA10g18580S*	Up stream	A10	16851683	A	C	-2.97	––
	*BjuA10g18580S*	Up stream	A10	16851690	G	A	-2.97	––
	*BjuA10g18950S*	Up stream	A10	17073204	A	T	-1.91	PF01026:TatD related DNase; PF00651:BTB/POZ domain|PF03000:NPH3 family
	*BjuA10g18960S*	Non synonymous	A10	17075651	A	G	-2.90	PF01722:BolA-like protein
	*BjuA10g23440S*	Non synonymous	A10	19294411	G	C	-5.45	PF00072:Response regulator receiver domain; PF00512:His Kinase A(phosphoacceptor)domain; PF02518:Histidine kinase,DNAgyraseB,and HSP90 like ATPase
	*BjuA10g23440S*	Non synonymous	A10	19294420	T	A	-5.45	same as above
	*BjuA10g23440S*	Non synonymous	A10	19294448	G	C	-5.45	same as above
	*BjuA10g24560S*	Down stream	A10	19829498	A	G	-3.33	PF03171:2OG-Fe(II) oxygenase superfamily; PF14226:non-haem dioxygenase in morphine synthesis N-terminal
InDels	*BjuA05g24310S*	Up stream	A05	23019187	AAAA	–	-4.58	PF13621:Cupin-like domain
	*BjuA05g24310S*	Up stream	A05	23019190	–	–	-4.58	same as above
	*BjuA10g16420S*	Up stream	A10	15695620	A	–	-4.69	PF03447:Homoserine dehydrogenase, NAD binding domain; PF00742:Homoserine dehydrogenase

– and –– mean no data detected. Log_2_FC is the mean of Log_2_FC at six comparison groups.

## 4 Discussion

Seed weight is one of yield components in *B. juncea*. Development of seed weight and seed size is a precise and complex regulated process and controlled by cues from maternal and zygotic tissues with some environmental influence (Li and Li, 2016). A recombinant inbred line (RIL) population was used to decrease the environmental influence in mapping QTLs for thousand grain weight (TGW) in wheat (Tahmasebi et al., 2017; Heidari et al., 2011). Identification of QTLs related to seed weight and/or size will contribute to improvement in crop yield. At present, the genomic regions and/or candidate genes regulating seed size in *B. juncea* have been identified using marker-based bi-parental quantitative trait loci (QTL) mapping or genome-wide association studies (Dhaka et al., 2017; Kang et al., 2021; Kumar and Bisht, 2020; Ramchiary et al., 2007; Sra et al., 2019). In addition, Namrata and his colleagues performed QTL analysis using *B. juncea* bi-parental doubled-haploid populations and identified 65 QTLs and numerous candidate genes influencing seed weight (Dhaka et al., 2017). However, more studies are needed to identify more genes for seed weight in *B. juncea.* In this study, we attempted to identify new candidate genes for TSW through BSA-seq of F_2_ progeny combined with transcriptome profiling of both parents 7981 and Sichuan Yellow.

BSA-seq is a method of constructing lineage populations containing extreme phenotypic traits, comparing their polymorphic loci by sequencing the parents and selected progeny with extreme trait values, and locating candidate intervals based on differences in allele frequencies (Zhang et al., 2018). We used this method in the present study and revealed chromosomes A01, A02, A03, A05, A07, A09, A10, B01, B02 and B04 probably carried the intervals for TSW in *B. juncea*. Consistent with the previous findings, the QTLs for TSW found by Namrata et al. were distributed on chromosomes A03, A07, A10 and B03 in *B. juncea* (Dhaka et al., 2017). The chromosomes A07, A08 and A09 contain highly significant QTLs for TSW in *B. rapa* (L.) (Alemu et al., 2024). In addition, the YLD-related QTLs were identified on chromosome A05 in *B. napus* (Ding et al., 2012). The candidate genes associated with seed yield in the candidate region of chromosome A02 were also identified in rapeseed (Pal et al., 2021). Therefore, the SNPs and Indels associated with TSW and corresponding chromosomal intervals detected in this study will provide a foundation for fine mapping for the genes involved in seed weight in *B. juncea*. Although the BSA method is commonly used for initial localization of genes with a relatively wide range and the bulked samples used in current study are not enough. The locations of TSW in *B. juncea* detected in current study also can further study for help breeding for TSW in *Brassica* combined with methods such as genetic mapping, molecular marker integration or amplified populations for further screening of genes within the range.

Among these candidate SNPs and InDels, seven SNPs with nonsynonymous mutations and three InDels were located in exons of annotated genes (**Table 3**). The *BjuA10g16450S*, with a SNP of nonsynonymous mutation, encodes Argonaute (AGO) protein possessing the PIWI-domain. The AGO protein is the central component of plant development, and its PIWI domain is required for target mRNA cleavage and therefore regulate the major processes in plant development (Mallory and Vaucheret, 2006; Baumberger and Baulcombe, 2005; Qi et al., 2005; Mallory et al., 2009). The new gene *BjuA10g22200S* identified in this study, whose exon has an InDel, encodes a CCAAT-binding transcription factor. The CCAAT-binding transcription factor controls various aspects of embryo and post-embryo development, and seed development and maturation in *Arabidopsis* (Tang et al., 2021; Tian et al., 2020). Moreover, subunits A, B, and C of the CCAAT-binding transcription factor increase the grain yield of wheat (Qu et al., 2015). The grain yield is always closely associated with TKW, and the QTLs for TKW were frequently associated with QTLs for grain yield (GY) in wheat (Shariatipour et al., 2021).The PPR repeat protein encoding genes, *BjuA10g20540S* and *BjuA10g14570S* harbored one candidate SNP with a nonsynonymous mutation and one candidate InDel with a frameshift insertion, respectively, The PPR repeat protein known as the PPR motif localized to mitochondria and/or chloroplasts and mediates diverse aspects of plant organelles and seed development in plants (Small and Peeters, 2000; Barkan and Small, 2014; Li et al., 2021; Schmitz-Linneweber and Small, 2008).Loss of function of PPR proteins usually leads to defects in embryogenesis and/or endosperm development and inhibits seed development (Li et al., 2014; Sosso et al., 2012). Deficiency of the mitochondrial P-type PPR protein EMP10 severely disturbs embryo and endosperm development, resulting in an empty pericarp or papery seeds in maize (Cai et al., 2017). Moreover, the P-type protein PPR5 was recently identified as a regulator required for endosperm development in rice, and *ppr5* mutants develop small starch grains (Zhang et al., 2019). These results showed that the PIWI domain, PPR repeat protein, and CCAAT-binding transcription factor-encoding genes related to the candidate SNPs/InDels identified in this study mostly participated in seed development, even in grain yield.

More than 7,000 DEGs were identified between the *B. juncea* accessions 7981 and the Sichuan Yellow at whole seed development stages. There are more DEGs at the S1 stage than at the other stages (**Figure 3A**). This is consistent with the study by Mathur and colleagues showing that more genes regulate seed development at the early seed stage in *B. juncea* (Mathur et al., 2022). Among the 7,679 DEGs common to the six comparison groups, 2,278 belonged to the 445 transcription factors (TF) family. Several DEGs were associated with the transcription factors Pkinase, RRM_1 and P450 (**Figure 3B**). The P450/CYP78A and P450/CYP79B1 had significantly higher expression in the high- than in low-yield rapeseed (*Brassica napus* L.) and are known to regulate seed yield by affecting anther and pollen development, seed ripening, seed size, and seed weight regulation (Salami et al., 2024). The P450/CYP78A gene family such as *AtCYP78A5*, *AtCYP78A6* and *AtCYP78A9* has been proven to be highly related to seed size in *Arabidopsis* (Wang et al., 2015). Moreover, transcriptome profiling has shown that cytochrome P450 superfamily protein-encoding genes are candidate genes associated with increased seed size in peanut (Liu et al., 2022c). Protein kinases and phosphatases are responsible for several cellular events mediated by protein phosphorylation and dephosphorylation. Among these events are cell growth and differentiation and cellular metabolism (MaChado et al., 2002). RRM is an RNA recognition motif and is abundant in RNA-binding proteins (RBPs). Gene Ontology enrichment analysis of barley RBPs indicated that these proteins were in all major cellular compartments and were associated with key biological processes, including translation, splicing, seed development and stress signaling (Mahalingam and Walling, 2020). In addition, disrupting the RNA binding activity of the RZ-1C protein, which contains RNA recognition motifs (RRMs), in *Arabidopsis* results in phenotypes that include delayed seed germination, reduced stature, and serrated leaves (Wu et al., 2016).

Yield is strongly correlated with biomass, which is mainly determined by photosynthetic efficiency (Heyneke and Fernie, 2018). The introduction of photorespiratory bypass agents into potato (*Solanum tuberosum*), *Camelina sativa*, and tobacco (*Nicotiana tabacum*) plants was found to increase photosynthesis, biomass yield, and tuber/seed yield (Dalal et al., 2015; Nölke et al., 2014). The photorespiratory bypass (GOC) related gene *OsGLO3* encoding glycolate oxidase 3 can significantly increases the photosynthesis efficiency and grain yield in rice (Shen et al., 2019; Wang et al., 2020). Our study showed that the expression of the glycolate oxidase 3 (FMN_dh)-encoding gene (*FMN_dh*) was upregulated in 7981 at six seed development stages (**Figure 3B**). Similarly, the glyoxalase-encoding genes (*BjuB03g21960S* and *BjuB04g27610S*) were also upregulated in 7981. Glyoxalase I (GLYI) and glyoxalase II (GLYII) play a vital role in the chemical detoxification of methylglyoxal (MG) in biological systems (Liu et al., 2022a). Overexpression of the glyoxalase gene *OsGlyI* improved abiotic stress tolerance and grain yield in rice (Zeng et al., 2016). One study suggested that *OsGLYI3* is specifically expressed in rice seeds and may be effective in promoting natural aging and longevity (Liu et al., 2022a). Metallothioneins (MTs) are polypeptide-encoded genes involved in plant growth, development, seed formation, and diverse stress responses (Liu et al., 2022b). One study revealed that the gene encoding metallothionein in maize may be regulated by abscisic acid (ABA) (Niu et al., 2022). During seed germination, storage proteins are decomposed to provide raw materials and energy for seedling growth. Metallothionein reduces the disulfide bond density of storage proteins, but increases their solubility and contribute to their mobilization (Aspart et al., 1984). The metallothionein-encoding genes (*BjuA02g40930S*, *BjuA05g34100S*, *BjuB01g04670S*, and *BjuB05g36620S*) were upregulated in 7981. From these results, we speculated that the genes encoding glyoxalate oxidase 3, glyoxalase and metallothionein may affect the difference in seed phenotype between the two *B. juncea* accessions investigated.

Nine genes were found to be both candidate SNP/InDel-located genes and differentially expressed genes between the two accessions at each seed development stages. The annotation showed that these genes encode the 2OG-Fe(II) oxygenase superfamily protein, BTB/POZ domain, cupin-like domain, homoserine dehydrogenase, BolA-like protein and histidine kinase. The 2OG-Fe (II) dioxygenase superfamily is the second largest protease family in plants and the genes encoding 2OG-Fe(II) dioxygenase GA2ox, GA3ox, and GA20ox are the key enzymes involved in gibberellin (GA) biosynthesis and play fundamental roles in plant growth and development (Ding et al., 2020; Hedden and Thomas, 2012; Han and Zhu, 2011). In addition, the gene *AHT1* encoding ABA-HYPERSENSITIVE BTB/POZ PROTEIN 1, which contains a BTB/POZ domain, was upregulated more than 2.5 times by abscisic acid (ABA) in *Arabidopsis*, and the loss of AHT1 led to retardation of the germination process (Kim et al., 2016). Although few reports have demonstrated that the 2OG-Fe (II) oxygenase and BTB/POZ domain-containing genes directly affect seed weight and size, the regulation of these two genes during seed development deserves further study because of their important role in plant growth.

In the present study, the histidine kinase- and BolA-like protein encoding genes *BjuA10g23440S* and *BjuA10g18960S* were not only DEGs but also candidate SNP-related genes, and the SNPs in two genes were nonsynonymous (**Table 3**). The plant phytohormone cytokinin is a key growth regulator involved in the regulation of a wide range of developmental processes, including root and shoot growth, photomorphogenesis, flowering timing, senescence, seed development, and even crop yield (Kim et al., 2016). The histidine kinase is the key components of the phosphorelay in cytokinin signaling (Mok and Mok, 2001). *Arabidopsis* His-kinases (AHKs) were shown to play redundant or specific roles in the regulation of root and shoot growth, leaf aging and seed size (Riefler et al., 2006). QTL mapping found one major QTL for TSW, cqSW.A03-2 in *B. napus*. The histidine kinase gene (*BnaA03G37960D*) is likely to be a candidate gene for cqSW. A03-2 locus (Wang et al., 2020). Interestingly, consistent with the above study, the histidine kinase-encoding gene *BjuA10g23440S* identified in our study was also a candidate TSW gene in *B. juncea*. BolA-like proteins are ubiquitous and are present in numerous organisms, ranging from bacteria to higher eukaryotes (Qin et al., 2015). However, the physiological function of BolA proteins in plants has yet to be elucidated. The *bola3* mutant does not exhibit any notable phenotype under normal growth conditions but rather grows better than the wild type under some stresses (Qin et al., 2015). The BolA-like protein-encoding gene *BjuA10g18960S* was also a putative candidate gene related to TSW as shown in this study. The regulatory effect of BolA-like proteins on seed weight is worthy of further study.

## 5 Conclusions

Ten regions associated with TSW were identified on chromosomes A01, A02, A03, A05, A07, A09, A10, B01, B02 and B04 by BSA-seq. Thirty five candidate SNPs and fifty eight candidate InDels were located in thirty two and fifty one genes, respectively. Candidate SNP/InDel-related genes containing the PIWI domain, the pentatricopeptide repeat (PPR) protein and the CCAAT-binding transcription factor-encoding genes were identified and may participate in the regulation of seed development. The DEGs, including the Pkinase, RRM_1 and P450 TF families, were identified, while the genes encoding glyoxalate oxidase 3, glyoxalase and metallothionein were found to be significantly upregulated in the heavy-seed line 7981 at each seed development stages. Combined with RNA-seq and comparative transcriptome analysis of both parents contrasting in seed weight, nine candidate genes for TSW were identified, among which the histidine kinase- and the BolA-like protein-encoding genes *BjuA10g18960S* and *BjuA10g23440S* are most likely candidate genes for seed weight in *B. juncea*. Our study paves a way for cloning of genes for seed weight and marker-assisted selection for bold seed in *B. juncea*.

## Data Availability

The datasets of BSR and BSA transcriptome data generated during the current study are available in NCBI, Accession number: PRJNA1020652 and PRJNA1022584.
