# Supplementary material for: Integrated analysis of BSA-seq and RNA-seq identified the candidate genes for seed weight in Brassica juncea
Source: Front Plant Sci. 2024 Dec 3;15:1458294. doi: 10.3389/fpls.2024.1458294 (PMC11654836; doi:10.3389/fpls.2024.1458294)
Supplement: Supplementary file 1 [file DataSheet1.zip › supplementary materials/supplementary materials.docx]

**Supplementary materials**

**Supplementary tables**

**Table S1.** The primers used in the present study for validation RNA-Seq.

| **Transcript_id** | **Primer name**  **(Sense/Anti-sense primer)** | **Nucleotide sequence** |
| --- | --- | --- |
| BjuA10g15340S | F | 5' CGACGGCTGGAACATACTCG 3' |
| BjuA10g15340S | R | 5' CATCCAGCCCACCAAGAACC 3' |
| BjuA10g18580S | F | 5' CTTCGCCAGAAGACGACGAC 3' |
| BjuA10g18580S | R | 5' TCGACGAACACCCAAAACCCA 3' |
| BjuA10g23440S | F | 5' AGCCAGACGACACAACCGAA 3' |
| BjuA10g23440S | R | 5' CGGCGACAGAACCGTTGATG 3' |
| BjuA10g18960S | F | 5' CTTCGTCTCCTGCTCCGATCT 3' |
| BjuA10g18960S | R | 5' TGGTGGACTCTCCACGAGAC 3' |
| BjuA02g40930S | F | 5' GGGCTTCTCAGGGGAGACTA 3' |
| BjuA02g40930S | R | 5' TCCGCACTTGCATCCATCAT 3' |

**Table S2.** The statistic of sequencing data in bulk segregant analysis sequencing (BSA-Seq).

| **Sample** | **Raw bases (bp)** | **Clean bases (bp)** | **Q20 (%)** | **Q30 (%)** | **GC content (%)** |
| --- | --- | --- | --- | --- | --- |
| **7981** | 12,151,542,300 | 11,890,035,000 | 97.44 | 92.89 | 38.28 |
| **Sichuan Yellow** | 13,534,605,000 | 13,375,162,500 | 97.33 | 92.62 | 38.06 |
| **LS_bulk** | 48,183,116,700 | 47,564,351,100 | 96.31 | 90.22 | 38.28 |
| **SS_bulk** | 53,084,066,700 | 52,346,697,900 | 97.47 | 93.24 | 39.75 |
| **Total** | 126,953,330,700 | 125,289,937,200 | -- | -- | -- |

**Table S3.** The SNP and InDel detection and annotation.

| **SNP information** | | | | | |
| --- | --- | --- | --- | --- | --- |
| **Category** | **7981** | **LS_bulk** | **Sichuan**  **Yellow** | **SS_bulk** | **Number of SNPs** |
| **Exonic** | 527,792 | 532,628 | 4,301 | 536,905 | 576,970 |
| **Exonic Stop gain** | 3,077 | 3,142 | 54 | 3,190 | 3,715 |
| **Exonic Stop loss** | 762 | 771 | 10 | 771 | 842 |
| **Exonic Synonymous** | 313,928 | 316,276 | 2,183 | 318,595 | 338,742 |
| **Exonic Non-synonymous** | 207,853 | 210,266 | 1,970 | 212,163 | 230,855 |
| **Exonic unknowns** | 2,172 | 2,173 | 84 | 2,186 | 2,816 |
| **Intronic** | 605,573 | 620,518 | 4,530 | 625,745 | 689,888 |
| **Splicing** | 1,508 | 1,516 | 11 | 1,541 | 1,700 |
| **Upstream** | 323,488 | 335,060 | 2,321 | 334,578 | 373,715 |
| **Downstream** | 274,367 | 281,939 | 2,168 | 281,814 | 316,558 |
| **upstream/downstream** | 62,044 | 63,907 | 335 | 63,838 | 70,123 |
| **Intergenic** | 2,024,670 | 2,173,030 | 107,390 | 2,164,128 | 2,678,212 |
| **Others** | 86,849 | 87,308 | 382 | 87,725 | 93,962 |
| **Total** | 3,906,289 | 4,095,904 | 121,438 | 4,096,272 | 4,801,126 |
|  |  |  |  |  |  |
| **InDel information** | | | | | |
| **Category** | **7981** | **LS_bulk** | **Sichuan**  **Yellow** | **SS_bulk** | **Number of InDels** |
| **Upstream** | 109,624 | 118,104 | 1,994 | 118,805 | 132,221 |
| **Exonic Stop gain** | 872 | 944 | 6 | 1,021 | 1,300 |
| **Exonic Stop loss** | 129 | 125 | 2 | 132 | 144 |
| **Exonic Frameshift deletion** | 9,576 | 9,746 | 149 | 9,923 | 11,159 |
| **Exonic Frameshift insertion** | 8,472 | 8,741 | 87 | 8,932 | 10,136 |
| **Exonic Non-frameshift deletion** | 7,013 | 7,190 | 90 | 7,244 | 7,868 |
| **Exonic Non-frameshift insertion** | 6,689 | 6,904 | 34 | 6,987 | 7,551 |
| **Intronic** | 236,393 | 248,448 | 3,681 | 253,099 | 276,368 |
| **Splicing** | 1,852 | 1,891 | 37 | 1,937 | 2,088 |
| **Downstream** | 81,740 | 87,736 | 1,473 | 88,387 | 98,580 |
| **Upstream/Downstream** | 25,330 | 26,840 | 364 | 27,103 | 29,679 |
| **Intergenic** | 326,168 | 372,811 | 16,211 | 372,495 | 441,519 |
| **Others** | 40,337 | 41,591 | 589 | 42,030 | 45,362 |
| **Total** | 854,151 | 931,018 | 24,706 | 938,035 | 1,063,911 |

**Table S4.** The statistic of RNA-Seq data at six seed development in two lines of *Brassica juncea*.

| **Sample** | **Raw reads** | **Clean reads** | **Clean base** | **Q20** | **Q30** | **GC content** | **Total map (%)** |
| --- | --- | --- | --- | --- | --- | --- | --- |
| Sichuan Y-S1-1 | 44519046 | 43316596 | 6.5G | 98.38 | 94.84 | 46.14 | 42147242(97.3%) |
| Sichuan Y-S1-2 | 46760732 | 45679616 | 6.85G | 98.23 | 94.5 | 46.24 | 43895161(96.09%) |
| Sichuan Y-S1-3 | 46790492 | 46301092 | 6.95G | 98.31 | 95.12 | 46.32 | 44535799(96.19%) |
| Sichuan Y-S2-1 | 50904918 | 50317914 | 7.55G | 97.89 | 93.95 | 45.89 | 36055417(71.66%) |
| Sichuan Y-S2-2 | 47679356 | 46581708 | 6.99G | 98.11 | 94.38 | 46.54 | 41563857(89.23%) |
| Sichuan Y-S2-3 | 47515068 | 46872712 | 7.03G | 98.14 | 94.5 | 45.83 | 33802223(72.11%) |
| Sichuan Y-S3-1 | 47149406 | 45760346 | 6.86G | 98.31 | 94.72 | 47.44 | 44681411(97.64%) |
| Sichuan Y-S3-2 | 46999112 | 45905760 | 6.89G | 98.36 | 94.83 | 46.88 | 44783408(97.56%) |
| Sichuan Y-S3-3 | 45714578 | 45214976 | 6.78G | 98.39 | 95.37 | 47.24 | 43863144(97.01%) |
| Sichuan Y-S4-1 | 47354176 | 46120368 | 6.92G | 98.23 | 94.57 | 47.84 | 44528952(96.55%) |
| Sichuan Y-S4-2 | 45359488 | 44130356 | 6.62G | 98.4 | 94.95 | 48.07 | 42387300(96.05%) |
| Sichuan Y-S4-3 | 43801292 | 43281258 | 6.49G | 98.52 | 95.62 | 48.08 | 41662295(96.26%) |
| Sichuan Y-S5-1 | 45516296 | 44216594 | 6.63G | 98.25 | 94.59 | 47.99 | 40575451(91.77%) |
| Sichuan Y-S5-2 | 45440328 | 44681760 | 6.7G | 98.36 | 95.17 | 47.94 | 41430610(92.72%) |
| Sichuan Y-S5-3 | 43656204 | 43152718 | 6.47G | 98.47 | 95.51 | 48.23 | 39891059(92.44%) |
| Sichuan Y-S6-1 | 47739496 | 46282798 | 6.94G | 98.34 | 94.83 | 48.98 | 44766473(96.72%) |
| Sichuan Y-S6-2 | 49908760 | 48969256 | 7.35G | 98.37 | 94.88 | 48.76 | 47147460(96.28%) |
| Sichuan Y-S6-3 | 54964382 | 54498018 | 8.17G | 98.35 | 95.25 | 49.2 | 51851002(95.14%) |
| 7981-S1-1 | 41260514 | 40710228 | 6.11G | 98.21 | 94.89 | 45.75 | 35637900(87.54%) |
| 7981-S1-2 | 41260514 | 40710228 | 6.11G | 98.21 | 94.89 | 45.75 | 35637900(87.54%) |
| 7981-S1-3 | 41260514 | 40710228 | 6.11G | 98.21 | 94.89 | 45.75 | 35637900(87.54%) |
| 7981-S2-1 | 48052090 | 46227648 | 6.93G | 98.26 | 94.62 | 46.96 | 40758140(88.17%) |
| 7981-S2-2 | 47581012 | 45552798 | 6.83G | 98.18 | 94.41 | 47 | 39125235(85.89%) |
| 7981-S2-3 | 41128710 | 40645300 | 6.1G | 98.36 | 95.14 | 47.03 | 35632489(87.67%) |
| 7981-S3-1 | 46901794 | 46294996 | 6.94G | 98.31 | 94.79 | 47.31 | 41785346(90.26%) |
| 7981-S3-2 | 45857344 | 45018914 | 6.75G | 97.71 | 93.35 | 47.53 | 40095313(89.06%) |
| 7981-S3-3 | 45375088 | 44960672 | 6.74G | 98.29 | 95.08 | 47.27 | 40237328(89.49%) |
| 7981-S4-1 | 47053972 | 45442222 | 6.82G | 98.42 | 95.03 | 47.75 | 40358559(88.81%) |
| 7981-S4-2 | 45546308 | 43731988 | 6.56G | 98.37 | 95.03 | 47.62 | 37354861(85.42%) |
| 7981-S4-3 | 44145870 | 43394292 | 6.51G | 98.31 | 94.86 | 47.76 | 37904819(87.35%) |
| 7981-S5-1 | 48306144 | 47760972 | 7.16G | 98.11 | 94.3 | 48.44 | 43761552(91.63%) |
| 7981-S5-2 | 52160534 | 51567592 | 7.74G | 97.32 | 92.7 | 48.66 | 46711221(90.58%) |
| 7981-S5-3 | 41407626 | 40656048 | 6.1G | 98.55 | 95.68 | 48.33 | 37140321(91.35%) |
| 7981-S6-1 | 46754440 | 45412986 | 6.81G | 98.38 | 94.87 | 48.13 | 41651307(91.72%) |
| 7981-S6-2 | 46480368 | 45304858 | 6.8G | 98.14 | 94.38 | 48.29 | 41473447(91.54%) |
| 7981-S6-3 | 43621548 | 43230882 | 6.48G | 98.49 | 95.5 | 48.44 | 39651736(91.72%) |
| **Total** | 1661927520 | 1628616698 | 244.29G | - | - | - | - |
| **Mean** | 46164653 | 45239353 | 6.79G | 98.26 | 94.78 | 47.43 | 90.89% |

**Table S5.** The information of differentially expressed genes (DEGs) in six compared groups.

| **Compared groups** | **All DEGs** | **Up-regulated DEGs** | **Down-regulated DEGs** |
| --- | --- | --- | --- |
| 7981_S1 -vs.- Sichuan Yellow_S1 | 44,176 | 19,473 | 24,703 |
| 7981_S2 -vs.- Sichuan Yellow_S2 | 33,478 | 17,512 | 15,966 |
| 7981_S3 -vs.- Sichuan Yellow_S3 | 29,078 | 13,366 | 15,712 |
| 7981_S4 -vs.- Sichuan Yellow_S4 | 27,678 | 12,701 | 14,977 |
| 7981_S5 -vs.- Sichuan Yellow_S5 | 29,421 | 13,835 | 15,586 |
| 7981_S6 -vs.- Sichuan Yellow_S6 | 35,581 | 15,972 | 19,609 |
